# More than colour attraction: behavioural functions of flower patterns

**DOI:** 10.1016/j.cois.2015.09.005

**Published:** 2015-12

**Authors:** Natalie Hempel de Ibarra, Keri V Langridge, Misha Vorobyev

**Affiliations:** 1 University of Exeter, Centre for Research in Animal Behaviour, Department of Psychology, Exeter, UK; 2 University of Auckland, School of Optometry and Vision Science, Auckland, New Zealand

## Abstract

Flower patterns are thought to influence foraging decisions of insect pollinators. However, the resolution of insect compound eyes is poor. Insects perceive flower patterns only from short distances when they initiate landings or search for reward on the flower. From further away flower displays jointly form larger-sized patterns within the visual scene that will guide the insect's flight. Chromatic and achromatic cues in such patterns may help insects to find, approach and learn rewarded locations in a flower patch, bringing them close enough to individual flowers. Flight trajectories and the spatial resolution of chromatic and achromatic vision in insects determine the effectiveness of floral displays, and both need to be considered in studies of plant–pollinator communication.

## Highlights

• Insects perceive separately chromatic and achromatic aspects of colour patterns.
• Flowers present colour patterns as individual or shared displays.
• Visual appearance of flowers changes considerably with viewing distance.
• Pollinators use close-up views for landing and handling of flowers.
• Further away shared displays within the visual scene guide approach trajectories.

**Current Opinion in Insect Science** 2015, **12**:64–70

This review comes from a themed issue on **Neuroscience**

Edited by **Yehuda Ben-Shahar**

For a complete overview see the Issue and the Editorial

Available online 20th October 2015

**http://dx.doi.org/10.1016/j.cois.2015.09.005**

2214-5745/© 2015 The Authors. Published by Elsevier Inc. This is an open access article under the CC BY license (http://creativecommons.org/licenses/by/4.0/).

## Introduction

**Figure 1:**
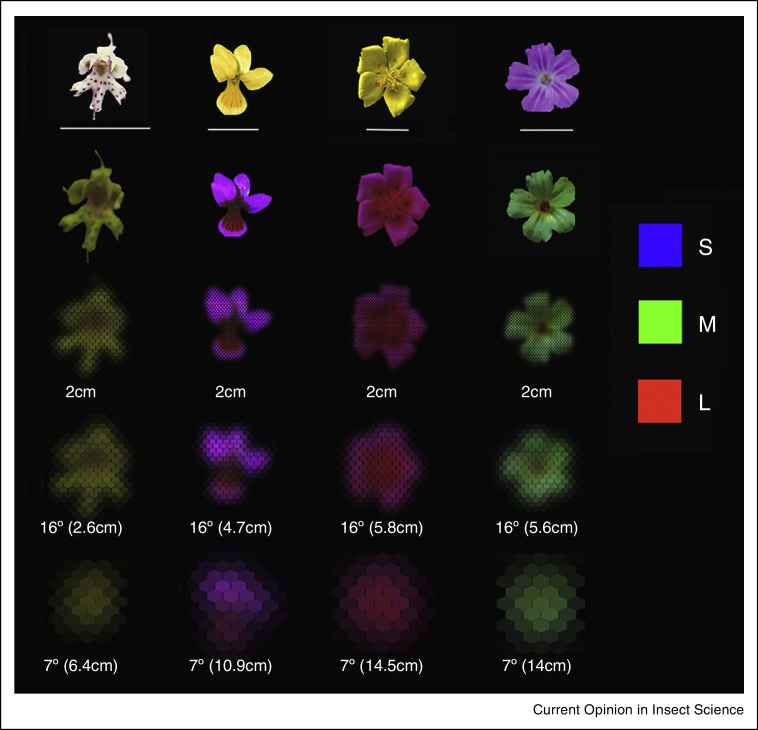
Flowers through bee eyes. Shown are pattern displays of small flowers (1 cm scale) in human colours (first row) and ‘bee colours’ (second row, high spatial resolution), for methods see [2, 29]. From left to right: *Traunsteinera globosa, Viola biflora, Helianthemum nummularium, Geranium robertianum.* Spectral sensitivities of the S, M and L-receptors of honeybees (peak sensitivities 344 nm, 436 nm, 556 nm) were used to calculate quantum catches in each pixel of the multispectral images. To show ‘bee colours’ (second row) quantum catches were converted into RGB values for the three primary monitor colours (see legend). The third row shows the images of single flowers projected onto the ommatidial lattice of the honeybee eye at a close distance (2 cm). Images in the fourth and lowest row simulate views at distances where the flower subtends a visual angle of 16°, which is above the chromatic threshold, or 7°, which is below the chromatic threshold (and approximately at the detection limit, within the range of the achromatic (brightness) visual system). Note that above the chromatic threshold, at short distances, only larger-sized patterns are optically resolved. Visually contrasting small ornaments or flower parts are visible when the insect is already on the flower and invisible during its approach flight (shown here for a distance of 2 cm at which a bee prepares for landing).

Visual information is indispensable for insect pollinators to locate, choose and interact with flowers. However, insect vision is constrained by the poor optical resolution of their small compound eyes, which is about a hundred times lower than that of our single-lens eye [1]. Unlike single-lens eyes, which are able to focus on objects at different distances, insect eyes have the same angular resolution at far and close distances. Therefore, insects are unable to resolve spatial details of distant objects, though they can use vision at extremely close distances. Theoretical analysis of the optical resolution of insect eyes demonstrates that most flower patterns can be resolved only when the insect is millimetres away [2] (Figure 1). Hence small-sized flower patterns do not play a role when insects approach flowers from some distance, as spatial details simply cannot be optically resolved. Resolution of chromatic vision is predicted to be lower than the eye's optical resolution. Different spectral types of photoreceptors that contribute to colour coding are randomly located across the eye [3]. Hence, chromatic vision requires that signals from more than one ommatidium are integrated which reduces the resolution below the limit set by the optics of the eye [4].

Under dim light conditions the spatial and temporal resolution of insect vision decreases further in order to improve contrast sensitivity. Many nocturnal insects, such as moths and beetles, have compound eyes with superposition optics, which confer higher sensitivity but lower spatial resolution than the apposition eyes of most diurnal insects. Several species of night-active bees are special in possessing diurnal-type apposition eyes with sufficient sensitivity to allow visually-guided foraging in twilight, and even during the night [5^••^]. The contrast sensitivity of such eyes can be enhanced by neural mechanisms, and anatomical evidence suggests that nocturnal bees sum signals from many ommatidia, albeit with the necessary reduction in spatial resolution [6]. Vision becomes slower under low light levels, due to temporal summation of receptor and neural signals that can occur in both types of eyes, and affect the insect's flight speed and trajectories [7, 8, 9••]. Interestingly, some nocturnal insects have not sacrificed colour vision in order to increase their visual sensitivity and can identify flowers on the basis of their colours even during moonless nights [10, 11].

Insect views of flowers differ fundamentally from ours, and human observers usually overestimate the signalling distance range and functions of floral displays [e.g. 12]. The low spatial resolution of insect eyes defines their perception of flower colours, shapes and patterns. Behavioural experiments confirm that insects cannot resolve small objects or small-scaled variations of shapes and patterns over long distances. For instance, the detection limit for single-coloured discs is 5° of angular size in honeybees, around 2° in large-sized bumblebees and 1° in swallowtail butterflies, which can be related to differences in eye size [13, 14, 15•]. For a 1 cm flower, this corresponds to a viewing distance of 11–57 cm, respectively. Dissectedness of the outline shape in flower-like targets impairs the detection range [16], as predicted by the optical model of the honeybee eye. The behavioural resolution of chromatic vision is even worse — honeybees cannot detect and discriminate targets on the basis of chromatic cues if they subtend a visual angle less than 13–15° [17, 18]. As viewing distances vary with an insect's movements, the appearance of flowers will change considerably, and consequently the insect must be able to rely on different visual cues when foraging and navigating in flower patches. To evaluate the functions of floral displays it is therefore not only important to know how they are resolved and processed by the visual system of an insect pollinator but to also consider an insect's flight trajectory at different distances from flowers.

## Why are flower patterns so widespread and diverse?

It is usually assumed that flower patterns increase the diversity of floral displays and help pollinators to discriminate between flowers and to identify the best-rewarding ones. However, when taking into account the poor resolution of compound eyes and typically small sizes of individual floral displays, it is evident that flower patterns can be seen by an insect and influence its behaviour only when it is already close to the flower, initiating a sequence of motor actions that lead up to landing and interactions with the flower. In that phase flowers can use patterns to exploit visuo-motor responses guiding an insect's movement [19, 20] to optimise pollen transfer and reduce potential damage from handling of the flower by the insect.

**Figure 2:**
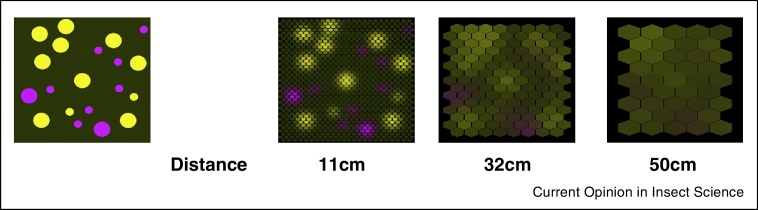
Shared floral displays through bee eyes. Shown is a simulated flower patch. The single-coloured target flower (1 cm in diameter) is in the centre. When the honeybee views the target flower from a distance of 11.4 cm it subtends a visual angle of 5°, the minimum angle to be detected. Its individual colour cannot be resolved at this distance. At a distance of 32 cm the target flower and other individual flowers in this patch are too small to be individually detected, but the whole group forms a shared display which subtends a visual angle of 15°. The mixed colour of this shared display will be visible to the approaching bee, but from further away (50 cm), it cannot be resolved. This patch and neighbouring groups of flowers form larger-sized patterns in the visual scene, with chromatic and brightness cues that can be used by the bee.

To communicate with insect pollinators over a distance, flowers must increase individual display sizes considerably or contribute to shared displays in inflorescences, mass displays or multi-species patches (Figure 2). Shared displays in a scene can produce effective signals with variable features, suited to influence the insect's approach behaviour when it moves through the environment, deciding where to go and which flowers to inspect and visit. Foraging decisions are not limited to the final stage of a floral visit. As the insect moves between flowers, the success of its foraging efforts is influenced by spatial memory processes and the cost of flight and interactions with flowers [21, 22, 23], and thus also by the effective visual guidance of the pollinator's movements. It is therefore important to consider the spatial scales, over which flower signals engage with visual and learning mechanisms, to understand the selective pressures that insect behaviour exerts on colour and pattern features of floral displays.

## Chromatic and achromatic processing in insect vision

The perception of colour patterns depends on the spatial distribution of contrast edges in an individual or shared display. These are processed by colour-blind edge detection and pattern discrimination mechanisms [24] that are segregated from a low-resolution chromatic system in insect vision [25, 26••, 27]. Achromatic and chromatic neural pathways operate in parallel and process, respectively, high and low-frequency components of visual scenes and objects.

Repetitive elements in pattern design found across angiosperms [28] point towards evolutionary selection of feature-dependent functions that target visually-guided behaviours of insects. Such behaviours are mediated in different ways by chromatic and achromatic visual mechanisms. For example, many flowers display a concentric (or ‘bulls-eye’) pattern that consists of a central disc surrounded by a contrasting outer ring. Patterns that have a bright (for bees) outer ring surrounding a dim disc can be detected from further distances than those having a bright disc surrounded by an outer dim ring. It appears that flowers with a bright outer ring are more common and tend to be smaller than those having a bright central disc and dim outer ring, suggesting that this arrangement may have been selected by insect vision [29]. Nevertheless, the overall detectability of both types of concentric patterns is worse than that of single-coloured discs (see Box 1), which suggests that these patterns have not evolved to simply attract pollinators. Instead they may be effective for flight control and stabilisation during landing and direct the insect towards the centre of the flower that contains the nectar and pollen rewards.

It is well known that insects discriminate a wide range of patterns and shapes, from simple to complex, artificial and naturalistic patterns in objects or visual scenes [24, 30, 31]. After extensive training, bees can learn to perform difficult tasks such as pattern grouping and categorisation [32]. Pattern vision is predominantly mediated by achromatic mechanisms; in bees by the L (long-wavelength sensitive or ‘green’) photoreceptor [e.g. 24]. Motion vision in insects is also colour-blind. Movement-derived visual information helps the insect to avoid collisions, negotiate narrow gaps, land on a surface, or locate the nest and foraging sites [recently viewed by^33^]. Motion parallax and looming cues can improve the detection range for an object placed in front of a background [34], facilitate landing manoeuvres at flowers with shapes of distinct depths, or positioning of the proboscis [35].

Movement causes motion blur, but its effect on pattern vision is negligible in visual systems that acquire visual information by fixating on objects. Although theoretically, it is plausible that insects reconstruct the image from temporal variations of the signal caused by motion, insects, such as flies and bees, fixate on objects, that is, acquire visual information in a similar way to vertebrates. To stabilise gaze they control the orientation of their body, which sometimes can deviate from their flight direction, and display saccadic movements which include fast body turns when changing the direction of gaze. Gaze stabilisation is supported by head movements [36, 37]; however, these are minute and extremely fast as the mobility of the head is limited by the insect's morphology.

## Flight trajectories influence foraging responses and learning

Since gaze direction is closely coupled with body orientation in insects, the viewing conditions, for example, distances and directions, during approach and landing on flowers will strongly depend upon the flight behaviour and navigational decisions. Thus, flight trajectories influence the perception and learning of sensory information by insects. When foraging insects navigate, their routes and approach trajectories are largely determined by the availability of suitable visual cues [38, 39]. Insects can, to some degree, flexibly adjust their flight behaviour for solving navigational and spatial orientation tasks by actively acquiring specific visual cues for spatial learning [40, 41]. This flexibility is influenced by the cost of efficiently executing flight and landing movements. Flying insects obey the laws of aerodynamics, hence approach and landing manoeuvres during a flower visit require a number of well-coordinated actions [42^•^]. To initiate a landing sequence at short distance from the flower the flying insect has to adjust the height of the flight trajectory and reduce its speed significantly. It has to maintain a good balance of its body to withstand aerodynamic drag downwards [43]. Sophisticated motor mechanisms rely on visual guidance allowing the insect to land elegantly [44^••^], rather than to crash into a flower, which is not a trivial task as flowers often move [45].

Flowers exploit the tight connection between vision and flight trajectory throughout the different phases of the approach flight and landing sequence. For example, field observations commonly describe the strong directionality of bumblebees foraging on vertical inflorescences, starting at the bottom and moving upwards [22, 46]. Flower orientation varies, and vertically-presented flowers on slopes tend to adaptively face down-slope, receiving more visitation as they offer convenient petal orientation for landing of bees moving preferentially upwards [47]. Observations on flowers reveal that flower orientation influences the landing behaviour of pollinators [48^••^]. It is beneficial for flowers to guide pollinator movement in a way that enhances pollen transfer [49], and field observations suggest that small patterns (‘nectar-guides’) help pollinators to orient on flowers [50, 51, 52].

## Colour and multimodal learning at the flower

The presence of colour in flower patterns is often suggested to attract insects towards the flower based on innate colour preferences and reflexive feeding responses [52, 53]. However, experience may be equally if not more important: insect pollinators quickly learn positive associations between food rewards and colour cues [for reviews see^54^, ^55^, ^56^]. The ability to memorise and discriminate diverse colour and pattern cues is well established for many insect pollinators, and consequently flower choices are strongly influenced by the sensory experience acquired during foraging and previous flower visits [57, 58, 59, 60, 61, 62]. Once the insect arrives at the flower and is able to see and recognise the contrasting colours of pattern elements, chromatic cues are likely to reinforce the decision to finalise a landing sequence or to follow contrast contours. Some colour elements in flower patterns may however present little or no chromatic contrast to the insect eye (Figure 1), and examples are best found among orchids which evolved an extreme diversity of colour patterns to accurately manipulate the insect's movements at the flower for a single opportunity to deposit pollinia on a specific body part of the insect.

Whilst at the flower, insects may combine cues for multimodal guidance, such as sensory information provided by the shape of the surface, texture, odours, and electrostatic forces [63•, 64, 65, 66]. As visual patterns help to make landing and reward localisation on a flower easier (alone or in combination with multimodal cues), the perceived reward value will be enhanced and learning improved; and consequently pollinators will show preferences for flowers with patterns.

## Conclusions

Pollinating insects forage in a three-dimensional environment and look at flowers from different distances and directions. What they see depends on the spatial resolution of the compound eye and visual mechanisms that process object information, however, it is also influenced by their flight trajectories and viewing conditions. What they choose depends on their vision and visual learning capabilities and is strongly influenced by navigation and spatial learning mechanisms. It remains to be understood how decisions are made and behavioural responses coordinated at far and near distances, as a pollinator moves between flowers, approaches and visits them. The underlying neural mechanisms involve basic sensory and motor systems that are shared across different taxonomic groups of insects. A wide range of flower search and choice behaviours adopted by insects can be explained by mechanistic models that take into account constraints imposed by the optics of insect eyes and aerodynamics of insect flight, rather than by models based on the assumptions of higher order cognitive processing of visual information.

## References and recommended reading

Papers of particular interest, published within the period of review, have been highlighted as:

• of special interest
•• of outstanding interest

## Figures and Tables

**Figure 3:**
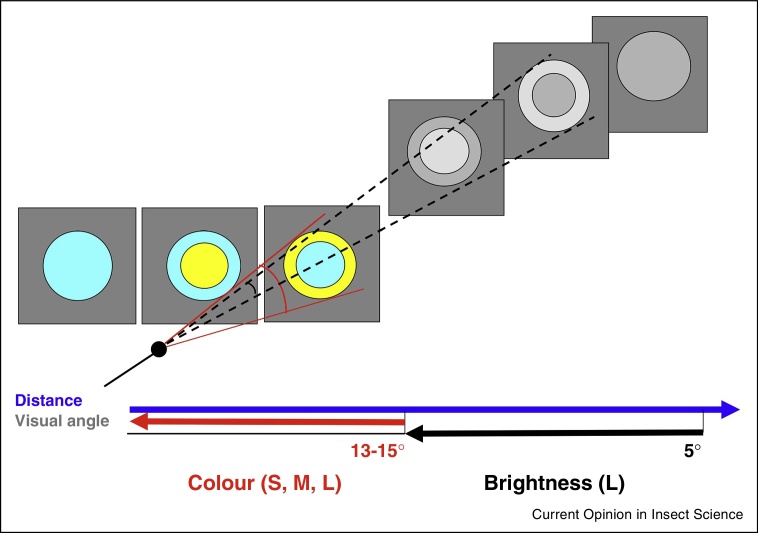
Spatial resolution of the honeybee's chromatic and achromatic visual system. Two parallel visual systems in the honeybee are tuned to objects of different sizes [13, 17, 18, 67]. At short distances when coloured discs subtend large visual angles, bees predominantly use chromatic cues to detect and discriminate coloured targets. The colour vision system receives input from all three receptor types (S, M, L). At longer distances, the achromatic visual system mediates detection and discrimination through the L-receptor contrast (achromatic or brightness contrast). The detection limit for a single-coloured disc presented individually is 5°. It does not vary with contrast strength. Signals from several adjacent ommatidia are processed, presumably by detector units with centre-surround receptive fields [68]. When the bee approaches the target, the angular size increases; above the chromatic threshold of 13–15° the target's colour will be resolved and chromatic cues determine the visual perception of bees. There is sensitivity for achromatic L-contrast but it is low; from short distances bees are able to detect very bright [69], but not less bright [68] achromatic discs. The distance range for detecting concentric patterns is shorter than for single-coloured discs of the same size and varies depending on the spatial arrangement of the pattern elements with different brightness contrasts (white – higher L-contrast, grey – lower L-contrast ).

## Box 1

Bees use colour (chromatic cues) to discriminate single-coloured discs and two-coloured concentric patterns, but only from close viewing distances (Figure 3). Bees are colour-blind while detecting and discriminating objects from further away, when those subtend small visual angles [13, 17, 18, 67]. In this case they rely on achromatic (brightness) cues, the signals of the L receptor alone. However, the detection threshold does not depend on the magnitude of L-receptor contrast. A critical parameter for the detectability of patterns is the distribution of L-receptor contrasts within the target [17, 67] (Figure 3). An outer ring with a strong L-receptor contrast (bright) surrounding a central disc with weak contrast (dim) is detected over a shorter distance than a single-coloured disc of the same colour and diameter. The detection distance is even shorter for patterns composed of a dim ring surrounding a bright disc [67]. When viewed through bee eyes such patterns have blurred edges; the impaired detectability is therefore likely to be a consequence of processing visual information by detector neurons with centre-surround organisation of their receptive fields [67]. These neurons are found in visual pathways of many animals. The consequences of detecting objects through such detectors vary for flowers of different sizes. Plants with smaller-sized flowers could have evolved compensatory strategies by sharing displays, without necessarily growing dense inflorescences or high densities of conspecific flowers. Sharing displays can also occur when different species grow in mixed patches next to each other (Figure 2), by offering large-sized visual features that inform an insect's navigational decisions and guide it towards a reward location with several flowers. In detection experiments honeybees and bumblebees showed a slightly improved detectability for groups of three discs that were placed at sufficiently large inter-disc distances to prevent optical merging when seen from a long distance [68]. This suggests that detector neurons interact to evoke differential responses towards extended distributions of objects across the visual scene.
